# Human carnosinase 1 overexpression aggravates diabetes and renal impairment in BTBR^Ob/Ob^ mice

**DOI:** 10.1007/s00109-020-01957-0

**Published:** 2020-08-15

**Authors:** Jiedong Qiu, Thomas Albrecht, Shiqi Zhang, Sibylle J. Hauske, Angelica Rodriguez-Niño, Xinmiao Zhang, Darya Nosan, Diego O. Pastene, Carsten Sticht, Carolina Delatorre, Harry van Goor, Stefan Porubsky, Bernhard K. Krämer, Benito A. Yard

**Affiliations:** 1 5th Medical Department, University Hospital Mannheim, Heidelberg University, Mannheim, Germany; 2 Department of Pathology and Medical Biology, University Medical Centre Groningen and University of Groningen, Groningen, The Netherlands; 3 Department of Endocrinology, The First Affiliated Hospital of Anhui Medical University, Hefei, China; 4 Central Medical Research Facility ZMF, University Hospital Mannheim, Heidelberg University, Mannheim, Germany; 5 Institute of Pathology, University Medical Center of the Johannes Gutenberg University Mainz, Mainz, Germany; 6 European Center for Angioscience, Mannheim, Germany

**Keywords:** Diabetic nephropathy, Carnosine, Antioxidants, Transgenic mice, Gene expression profiling

## Abstract

**Objective:**

To assess the influence of serum carnosinase (CN1) on the course of diabetic kidney disease (DKD).

**Methods:**

hCN1 transgenic (TG) mice were generated in a BTBR^Ob/Ob^ genetic background to allow the spontaneous development of DKD in the presence of serum carnosinase. The influence of serum CN1 expression on obesity, hyperglycemia, and renal impairment was assessed. We also studied if aggravation of renal impairment in hCN1 TG BTBR^Ob/Ob^ mice leads to changes in the renal transcriptome as compared with wild-type BTBR^Ob/Ob^ mice.

**Results:**

hCN1 was detected in the serum and urine of mice from two different hCN1 TG lines. The transgene was expressed in the liver but not in the kidney. High CN1 expression was associated with low plasma and renal carnosine concentrations, even after oral carnosine supplementation. Obese hCN1 transgenic BTBR^Ob/Ob^ mice displayed significantly higher levels of glycated hemoglobin, glycosuria, proteinuria, and increased albumin-creatinine ratios (1104 ± 696 vs 492.1 ± 282.2 μg/mg) accompanied by an increased glomerular tuft area and renal corpuscle size. Gene-expression profiling of renal tissue disclosed hierarchical clustering between BTBR^Ob/Wt^, BTBR^Ob/Ob,^ and hCN1 BTBR^Ob/Ob^ mice. Along with aggravation of the DKD phenotype, 26 altered genes have been found in obese hCN1 transgenic mice; among them claudin-1, thrombospondin-1, nephronectin, and peroxisome proliferator–activated receptor-alpha have been reported to play essential roles in DKD.

**Conclusions:**

Our data support a role for serum carnosinase 1 in the progression of DKD. Whether this is mainly attributed to the changes in renal carnosine concentrations warrants further studies.

**Key messages:**

- Increased carnosinase 1 (CN1) is associated with diabetic kidney disease (DKD).
- BTBR^Ob/Ob^ mice with human CN1 develop a more aggravated DKD phenotype.
- Microarray revealed alterations by CN1 which are not altered by hyperglycemia.
- These genes have been described to play essential roles in DKD.
- Inhibiting CN1 could be beneficial in DKD.

**Electronic supplementary material:**

The online version of this article (10.1007/s00109-020-01957-0) contains supplementary material, which is available to authorized users.

## Introduction

The global prevalence of type 2 diabetes is growing to epidemic proportions, affecting approximately 642 million adults by the year 2040 [1]. Approximately one-third of the patients with diabetes will develop DKD, making DKD the leading cause of chronic kidney disease (CKD) and end-stage kidney disease (ESKD) worldwide [2, 3].

Among the reported susceptibility loci for developing DKD, we identified serum carnosinase 1 (CN1, EC 3.4.13.20) and its substrate carnosine as modifiers of DKD. The possibility that carnosine may affect diabetic complications emerged from the finding that a trinucleotide (CTG)_n_ repeat polymorphism in the gene encoding CN1 was associated with susceptibility for developing DKD in type 2 diabetic patients [4, 5]. Other studies have confirmed this association [6–8], which seems to be stronger in females [7] than in male patients. The latter might be explained by lower serum CN1 activity/concentration generally found in male subjects [9].

It has been postulated that high serum CN1 expression may deplete tissue carnosine concentrations and render tissue more vulnerable to hyperglycemia mediated damage [10, 11]. Because serum CN1 concentrations are partly determined by the (CTG)_n_ polymorphism [5], this may explain the genetic association with DKD. However, a formal proof that serum CN1 directly affects the course of DKD is lacking. Human CN1 transgenic db/db mice developed a more severe diabetic phenotype compared to wild type db/db littermates, yet DKD did not differ between transgenic and wild-type mice [12]. Because db/db mice show only a mild renal phenotype of DKD, the influence of a disease modifier such as CN1 could have been masked. Even though a number of rodent studies already suggest a beneficial effect of carnosine supplementation on renal function impairment [12–17], more severe DKD models are warranted to better understand the role of the carnosine—carnosinase system in the progression of DKD. In the present study, we assessed the impact of serum CN1 expression on the course of DKD by generating hCN1 TG mice on a BTBR^Ob/Ob^ background and studying the development of obesity, diabetes, and renal impairment.

## Research design and methods

### Generation of transgenic mice

Human CNDP1 TG mice were generated in the BTBR^Wt/Ob^ (black and tan, brachyuric) background, as previously described [12]. The transgene TTP-hCNDP1 and the ob/ob mutation were genotyped by PCRs. Mice were bred from the initial founders to obtain the experimental groups.

Mice (*n* = 9–10 per group) were randomly allocated and were housed in a specific pathogen-free, regularly controlled animal house of the University Heidelberg at 22 ° C in a 12 h light/dark cycle and fed regular chow and water ad libitum. Starting with the 8th week of age, mortality, fasting plasma glucose, and body weight (BW) were recorded weekly in the morning until the 24th week of age. Glycated hemoglobin (HbA1c) percentage was measured every 8 weeks using the in2it A1C system (Bio-Rad Hercules, CA). At week 24 of age, blood samples were collected from the orbital plexus under anesthesia before sacrifice, and serum was isolated by centrifugation. To obtain morning spot urine samples, animals were placed in metabolic cages overnight.

### Anserine and carnosine concentrations

hCN1 TG BTBR^Wt/Ob^ and control BTBR^Wt/Ob^ mice (*n* = 6 per group) were supplemented with 4 mM carnosine in drinking water while drinking ad libitum. Non-TG and hCN1 TG BTBR^Wt/Ob^ mice served as controls (*n* = 7 and 6 per group). After 2 weeks, mice were sacrificed. Carnosine concentration was measured as previously described [18].

### Serum CN1 concentration and activity

CN1 concentrations in serum (*n* = 12 and 15 per group) were measured by a house-made sandwich ELISA as described previously [19]. CN1 activity was measured as previously described [18].

### Histology and immunohistology

Mice (*n* = 7–9 per group) were sacrificed at week 24 by vascular perfusion fixation through the aorta with 4% paraformaldehyde under ketamine/xylazine anesthesia. Right side kidneys were isolated afterward. Left side kidneys were snap-frozen and preserved before perfusion. Hereafter, all kidneys were weighed. Tissues fixed with paraformaldehyde were embedded in paraffin, cut in 2.5 μm sections, deparaffinized with xylol, and dehydrated using an ethanol gradient. Sections were stained with periodic acid-Schiff (PAS) and hematoxylin and eosin (H&E). Stained slides were digitalized using the PreciPoint M8 scanner, and area measurement was performed in ViewPoint software (both from Precipoint Freising, Germany). Per animal, a minimum of 30 glomeruli was analyzed. For mesangial matrix expansion, 20 glomeruli per animal were graded on PAS-stained sections using a scoring system: 0 for no, 1 for slight, 2 for moderate, and 3 for severe mesangial expansion.

For immune histology, sections were stained with rabbit polyclonal anti-CNDP1 antibody (ATLAS/Abcam Cambridge, UK), rabbit polyclonal anti-C3 antibody (Hycult HP8012), and Goat anti-rabbit HRP-conjugated IgG antibody (Santa Cruz, USA). Staining was visualized using red alkaline phosphatase (Vector Laboratories, USA) as a peroxidase substrate. The sections were counterstained with hematoxylin, dehydrated with a standard row of alcohol and xylol, then mounted.

### Urine parameters

Glucose, creatinine, and total protein in the urine of the animals (*n* = 6–10 per group) at week 22 after overnight metabolic cages were measured using a Cobas ® C311 autoanalyzer after 10 min centrifugation at 300 rpm to remove possible fecal contaminations. Albumin was determined by a competitive ELISA.

### Microarray

Total RNA (*n* = 5–7 per group) was prepared using TriZol (Thermo Fisher Scientific Karlsruhe, Germany) followed by additional purification using the RNeasy Mini Kit (Qiagen Hilden, Germany). RNA quality was assessed by capillary electrophoresis on an Agilent 2100 bioanalyzer. Only RNA samples with RIN values above 7.0 were used for further analysis. Gene expression profiling was performed using arrays of Mouse Gene 2.0 ST array from Affymetrix according to manufacturer’s protocols. All the equipment used was from the Affymetrix-Company (Affymetrix High Wycombe, UK).

### Bioinformatics

A Custom CDF Version 22 with ENTREZ based gene definitions was used to annotate the arrays [20]. The raw fluorescence intensity values were normalized, applying quantile normalization and RMA background correction. One-way ANOVA was performed to identify differentially expressed genes. A false-positive rate of *a* = 0.05 with FDR correction was taken as the level of significance.

### Statistics and figures

Data are depicted and described in the text as mean ± standard deviation. The experimental groups were compared using one-way ANOVA followed by Tukey’s post hoc test. The comparisons were performed two-tailed, and a *p* value below 0.05 was considered to be significant. GraphPad Prism version 8 for Windows (California USA) was used to create the figures.

## Results

### hCN1 expression in TG mice

**Fig. 1:**
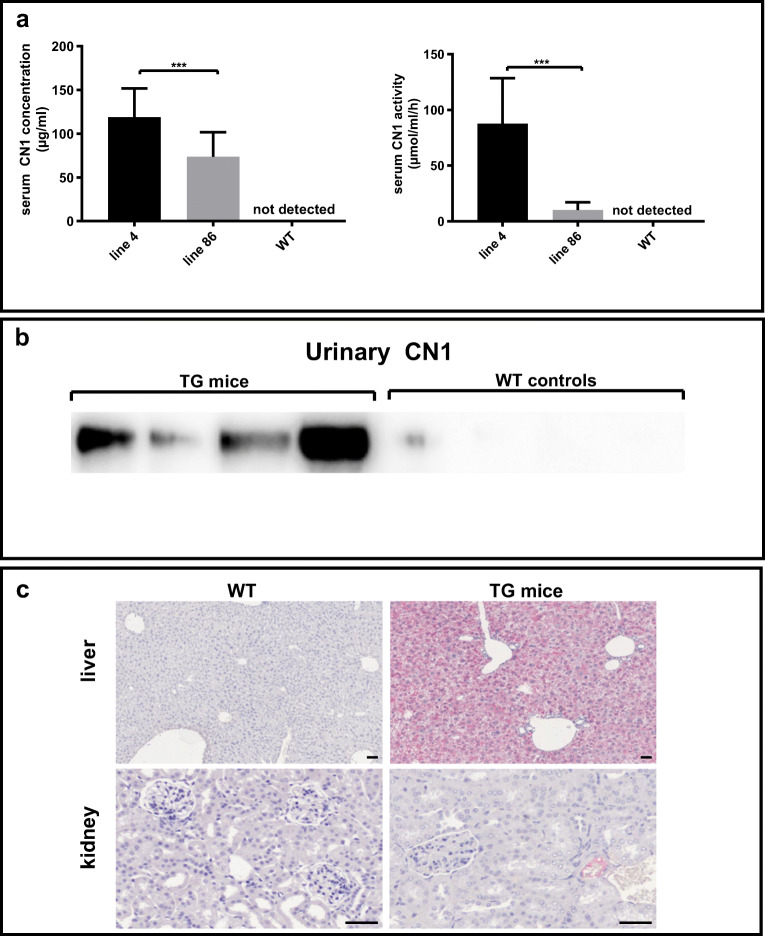
Two TG founder lines, i.e., line 4 and line 86, were studied for serum CN1 concentration and activity and urinary CN1 expression. **a** Serum CN1 concentration (panel to the left) and CN1 activity (***n*** = 12–18 per transgenic group) are depicted. The results are expressed as mean ± SD. ***T*** test was used to compare the line 4 and line 86. *** for ***p*** values < 0.001. **b** Western blot analysis for urinary CN1 expression from 4 WT and TG mice derived from founder line 4 is depicted. In **c**, immunohistochemistry of CN1 in CNDP1 transgenic (TG) and nontransgenic wildtype (WT) mouse shows positivity in liver parenchyma of CN1 TG mice (in the upper panel). In contrast, kidneys of CN1 TG mice showed only intravasal positivity, probably due to the serum carnosinase (in the lower panel). Scale bar: 50 μm

Two founder lines, i.e., line 4 and line 86, were generated that significantly differed in serum CN1 concentrations. In line 4, serum CN1 concentrations ranged from 51 to 171 μg/ml (*n* = 15), whereas TG mice derived from line 86 displayed lower CN1 concentrations (range: 18 to 98 μg/ml, *n* = 12) (*p* < 0.001) (Fig. 1a). Serum CN1 activity was likewise higher in TG mice of the former founder line (*p* < 0.001) (Fig. 1a). Urinary CN1 was detected in all TG mice derived from founder 4, albeit this largely varied between individual mice as detected by western blot (Fig. 1b). In contrast, urinary CN1 in TG mice from line 86 was low, i.e., slightly above or below the detection limit (data not shown). In immunohistochemistry, strong CN1 expression was detected in the liver but not in the kidney of CN1 transgenic mice of both lines (Fig. 1c).

### Depletion of carnosine

**Fig. 2:**
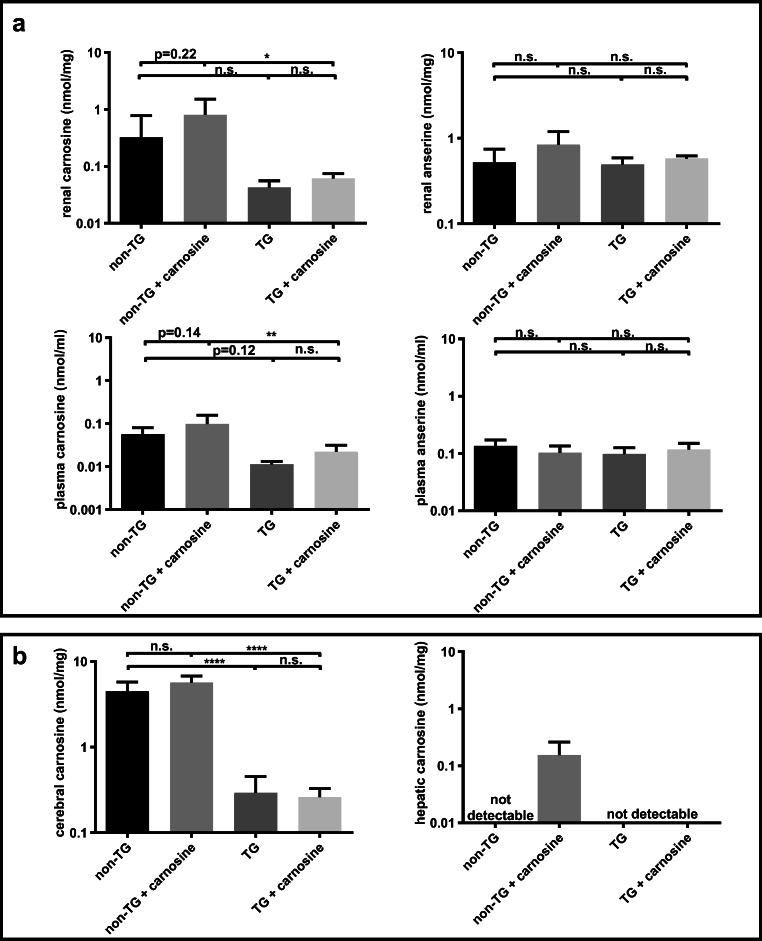
hCN1 transgenic (TG) and nontransgenic BTBR^Ob/Wt^ mice (nonTG) at the age of 10–14 weeks were either or not supplemented for 2 weeks with 4 mmol of carnosine in their drinking water (***n*** = 4–7 per group). a Plasma (upper panels) and renal (lower panel) carnosine and anserine concentrations were assessed. b Cerebral and hepatic carnosine concentrations were assessed. Mice from line 4 were used. Carnosine concentrations were measured using HPLC. The concentrations are denoted as nmol/mg and nmol/ml and shown as mean ± SD. One-way ANOVA followed by Tukey post hoc test was used to compare the groups. * for ***p*** values < 0.05, ** for ***p*** values < 0.01, *** for ***p*** values < 0.001 and n.s. for ***p*** values > 0.05

To assess to what extent CN1 expression affected CN1 substrates in renal tissue, subgroups of TG and non-TG BTBR^Ob/Wt^ mice (of founder line 4) were orally supplemented with 4 mM L-carnosine for 14 days and compared with nonsupplemented controls (Fig. 2). Although the mean renal carnosine concentrations were approximately 8-fold lower in TG mice derived from founder line 4 as compared with their nonTG littermates, this difference was not significant due to the high variance (*p* = 0.69). Renal anserine concentrations were not different between TG and nonTG mice.

After oral carnosine supplementation, there was a trend in nonTG mice towards increased renal carnosine and anserine concentrations, while in TG mice renal carnosine and anserine concentrations remained low after oral carnosine supplementation. Similar to renal carnosine concentrations, in plasma, there was a trend towards a lower carnosine concentration in TG mice as compared with wild-type mice.

In the brain tissue, the carnosine concentrations were significantly lower in TG mice as compared with nonTG littermates. In liver tissue, carnosine could only be detected after oral supplementation in nonTG mice, while it was not detected in the liver of TG mice, even after oral carnosine supplementation (detection limit at 0.001 nmol/mg) (Fig. 2).

### Influence of CN1 on the course of DKD

**Fig. 3:**
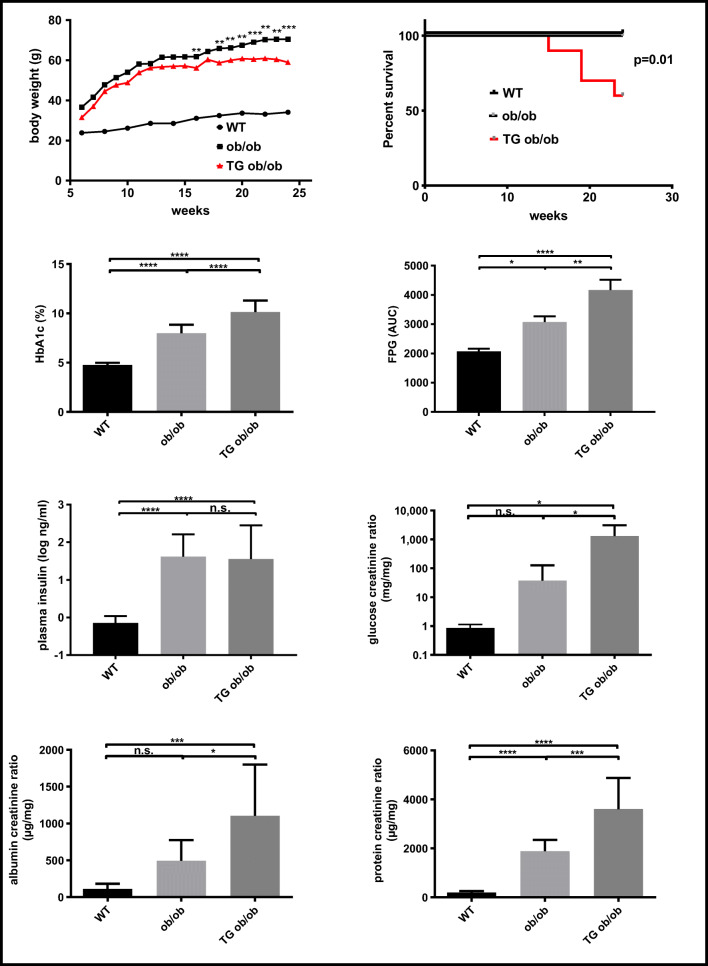
Wildtype BTBR^Ob/Wt^ (WT), nontransgenic BTBR^Ob/Ob^ (nonTG ob/ob) and transgenic BTBR^Ob/Ob^ (TG ob/ob) mice were observed for 18 weeks until the 24th week of age (***n*** = 6–9 per group). Body weight development, mortality, HbA1c, fasting plasma glucose (FPG), plasma insulin, glucosuria, albuminuria, and proteinuria are shown. All urinary parameters are shown as ratios to creatinine. The data are depicted as mean ± SD. One-way ANOVA followed by Tukey post-hoc test was used to compare the groups. Mantel-Cox-test was used to compare mortality (***n*** = 9–10 per group). * for ***p*** values < 0.05, ** for ***p*** values < 0.01, *** for ***p*** values < 0.001 and n.s. for ***p*** values > 0.05

Because of its higher serum CN1 concentration and activity, we employed TG and nonTG littermates derived from line 4 for all further experiments. Similar results were, however, generally replicated in a small subset of TG BTBR^Ob/Ob^ mice derived from line 86 (data not shown). Wildtype (WT) nondiabetic BTBR^Ob/Wt^, diabetic nonTG BTBR^Ob/Ob^ (ob/ob) and diabetic TG BTBR^Ob/Ob^ (TG ob/ob) were followed from week 6 to week 24 after birth. Compared with WT control, body weight significantly increased in both ob/ob groups (*p* < 0.0001). TG ob/ob mice developed a significantly lower body weight than nonTG ob/ob (*p* = 0.0036) (Fig. 3). A significantly increased mortality (4/10) was observed for hCN1 TG ob/ob mice after 18 weeks of observation (*p* = 0.01) (Fig. 3).

Serum glycated hemoglobin (HbA1c) levels were increased in TG compared with nonTG ob/ob mice by approximately 2% (21 mmol/mol) (*p* < 0.0001). Similarly, fasting plasma glucose (FPG) was increased in both diabetic groups compared with WT controls (*p* < 0.05), and TG ob/ob mice had significantly higher FPG than their nontransgenic diabetic siblings (*p* < 0.01). Plasma insulin significantly increased in both diabetic groups with no influence of the transgene herein (Fig. 3). Glycosuria was not significantly higher in nonTG ob/ob mice as compared with WT controls. However, in TG ob/ob mice, it was increased more than 30-fold as compared with nonTG obese controls (*p* = 0.01). Proteinuria, expressed as urinary protein-creatinine ratio, was significantly increased in both diabetic groups, approximately 2-fold higher in TG ob/ob as compared with the nonTG ob/ob group (*p* = 0.0002). Compared with WT controls, urinary albumin-creatinine ratio (ACR) was increased approximately 5-fold in ob/ob mice, albeit with a borderline significance of p = 0.09. In TG ob/ob mice, ACR was further increased and significantly differed from ob/ob mice (1104 ± 694 μg/mg vs 492.1 ± 282.2 μg/mg, *p* = 0.01).

**Fig. 4:**
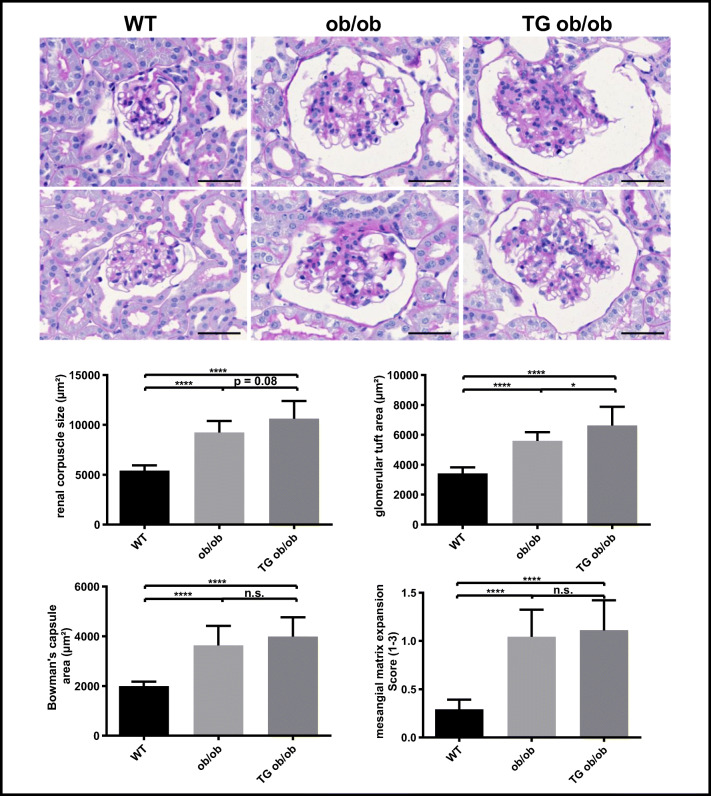
Kidneys from BTBR^Ob/Wt^ (WT), nontransgenic BTBR^Ob/Ob^ (ob/ob) and transgenic BTBR^Ob/Ob^ (TG ob/ob) were assessed (***n*** = 6–9 per group). Renal corpuscle size, tuft area, and Bowman’s capsule space were measured biometrically in > 30 renal corpuscles per animal. Mesangial matrix expansion was assessed using a score (0–3) in 20 renal corpuscles per animal. The data are depicted as mean ± SD. One-way ANOVA followed by Tukey post hoc test was used to compare the groups. * for ***p*** values < 0.05, ** for ***p*** values < 0.01, **** for ***p*** values < 0.0001 and n.s. for ***p*** values > 0.05. Scale bar: 50 μm

Diabetic animals revealed significant mesangial matrix expansion, enlarged renal corpuscles, glomerular tuft, and increased Bowman’s space. Differences between diabetic TG ob/ob and nonTG ob/ob were found for the glomerular tuft area (6623 ± 1257 μm^2^ vs 5594 ± 575.5 μm^2^, *p* = 0.04) and renal corpuscle size with borderline significance (10610 ± 1782 μm^2^ vs 9231 ± 1149 μm^2^, *p* = 0.08) (Fig. 4).

In plasma of diabetic animals, no significant difference in glyoxal and methylglyoxal concentrations were detected. Although diabetic hCN1 TG mice showed significantly higher (*p* < 0.01) 3-deoxyglucosone than their diabetic littermates, this was not significant compared with the nondiabetic WT controls (Supplementary data). In line with this, OxyBlot analysis of renal tissue gave no indication for increased protein carbonylation in diabetic vs non-diabetic animals (Supplementary data).

### Gene expression profiling of renal tissue

**Fig. 5:**
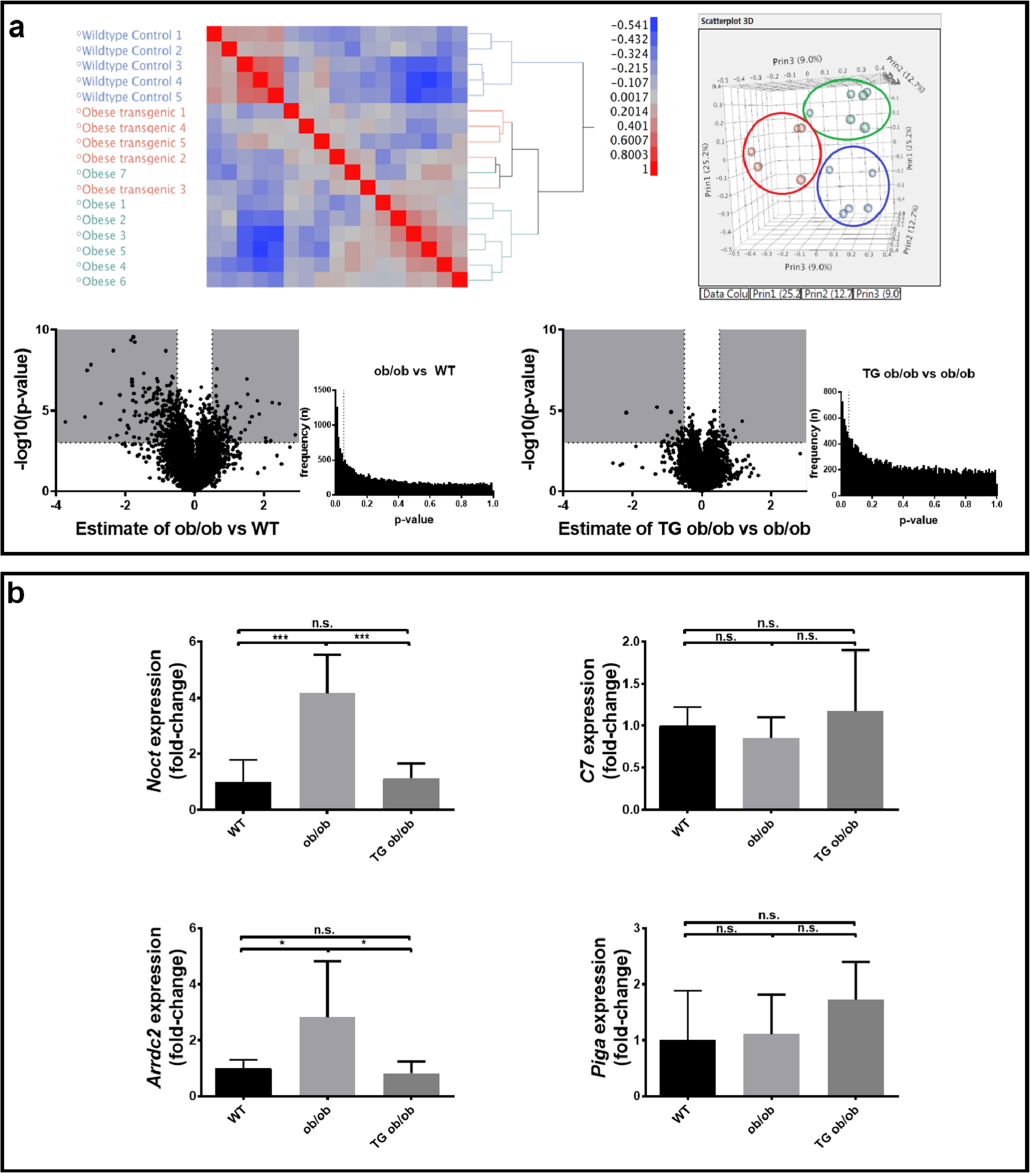
Gene expression profiling analysis in renal tissue from BTBR^Ob/Wt^ (WT), nontransgenic BTBR^Ob/Ob^ (nonTG ob/ob) and transgenic BTBR^Ob/Ob^ (TG ob/ob) was performed (***n*** = 5–7 per group). Hierarchical clustering analysis followed by a principal component analysis was performed to compare the similarity between the groups. Differently expressed genes are plotted in a volcano-plot where the estimate is their change in log^**2**^. An adjusted ***p*** value below 0.05 was considered as significant. ***P*** value distribution histograms are created using unadjusted ***p*** values. Ultimately, the altered expression of 4 genes (**Noct, C7, Arrdc2, and Piga**) could be confirmed by qPCR

To obtain more mechanistic clues why hCN1 TG mice displayed a more severe renal phenotype, we performed gene expression profiling of renal tissue retrieved from WT, hCN1 TG ob/ob, and nonTG ob/ob mice. Hierarchical clustering and principal component analysis disclosed distinct expression patterns between the groups (Fig. 5a). The difference in gene expression profile between diabetic ob/ob and WT mice was more profound as compared between the two diabetic subgroups, as shown by volcano plots and *p* value distribution (Fig. 5a).

By applying an adjusted *p* value < 0.05 (*P*_adj_ as adjusted for multiple testing) and a fold-change (FC) threshold of ≥ 1.5, a total of 297 transcripts were found to be differentially expressed in the comparison between ob/ob and WT. In Table 1, the 15 most upregulated and 15 most downregulated genes in the comparison ob/ob and WT mice are depicted. In kidneys of diabetic ob/ob mice, 3-hydroxy-3-methylglutaryl-coenzyme A synthase 2 (Hmgcs2) was found to be the strongest upregulated gene (7.4-fold upregulated), while histidine decarboxylase (Hdc) was the most downregulated gene as compared with their nondiabetic littermates (36-fold downregulated). Differences in mRNA expression for Noct, Hyal, Igf1, and C3 were confirmed by qPCR (Supplementary data). Immunohistochemistry for C3 was concordant to the qPCR results (Supplementary data).

**Table 1:** ob/ob vs WT

Gene symbol	Gene name	Fold change	*p* value
Top 15 most upregulated genes			
Hmgcs2	3-Hydroxy-3-methylglutaryl-Coenzyme A synthase 2	2.9086	2.95E-02
Noct	Nocturnin	2.4448	2.24E-03
Aldh1a7	Aldehyde dehydrogenase family 1, subfamily A7	2.2304	2.10E-03
Slc25a25	Solute carrier family 25 (mitochondrial carrier, phosphate carrier), member 25	2.1881	4.54E-02
8430408G22Rik	RIKEN cDNA 8430408G22 gene	2.0604	4.82E-02
Nat8f5	N-Acetyltransferase 8 (GCN5-related) family member 5	2.0502	3.72E-02
Clca3a1	Chloride channel accessory 3A1	1.8610	5.44E-03
C3	Complement component 3	1.8026	1.86E-03
Aldh1a1	Aldehyde dehydrogenase family 1, subfamily A1	1.6467	6.86E-03
Col8a1	Collagen, type VIII, alpha 1	1.5908	1.14E-02
Dpys	Dihydropyrimidinase	1.5328	1.20E-03
Gc	Group specific component	1.5069	3.68E-04
Ugt1a6b	UDP glucuronosyltransferase 1 family, polypeptide A6B	1.4530	2.07E-02
Inmt	Indolethylamine N-methyltransferase	1.3338	2.53E-02
Arrdc2	Arrestin domain containing 2	1.3095	2.94E-03
Top 15 most downregulated genes			
Hdc	Histidine decarboxylase	− 5.2181	1.49E-05
Serpina6	Serine (or cysteine) peptidase inhibitor, clade A, member 6	− 3.7336	1.09E-02
Slc22a7	Solute carrier family 22 (organic anion transporter), member 7	− 3.1630	7.05E-03
Akr1c18	Aldo-keto reductase family 1, member C18	− 3.1049	1.63E-04
Havcr1	Hepatitis A virus cellular receptor 1	− 2.9969	9.37E-05
Scd1	Stearoyl-coenzyme A desaturase 1	− 2.7010	2.22E-03
Akr1c14	Aldo-keto reductase family 1, member C14	− 2.4498	3.68E-02
Sec14l3	SEC14-like lipid binding 3	− 2.3448	1.49E-05
Abcc3	ATP-binding cassette, subfamily C (CFTR/MRP), member 3	− 2.2403	3.05E-02
Slc22a26	Solute carrier family 22 (organic cation transporter), member 26	− 2.1596	1.25E-03
Mep1b	Meprin 1 beta	− 2.1511	9.03E-03
Gbp3	Guanylate binding protein 3	− 2.0440	4.27E-03
Slc22a19	Solute carrier family 22 (organic anion transporter), member 19	− 1.9632	2.62E-02
Ace	Angiotensin I converting enzyme (peptidyl-dipeptidase A) 1	− 1.9385	1.48E-02
Slc22a29	Solute carrier family 22. member 29	− 1.8935	1.79E-02

Fold change is displayed as log^2^ value

For the comparison TG vs. nonTG ob/ob mice, only 26 transcripts were differentially expressed using the criteria defined above (Table 2). Complement factor 7 (C7) was the most upregulated gene (2.2-fold upregulated), and nocturnin (Noct) was the most downregulated gene (4.54-fold downregulated). Differences for Noct, C7, Arrdc2, and Piga between TG ob/ob and nonTG ob/ob were also confirmed by qPCR, albeit that not for all genes significance was reached (Fig. 5b).

**Table 2:** TG ob/ob vs ob/ob

Gene symbol	Gene name	Fold change	*p* value
11 upregulated genes			
C7	Complement component 7	1.1658	1.0468E-02
Egfl6	EGF-like-domain, multiple 6	0.9664	3.5986E-02
Cldn1	Claudin 1	0.9050	2.1179E-02
Tchhl1	Trichohyalin-like 1	0.7332	3.1491E-02
Thbs1	Thrombospondin 1	0.6836	4.3827E-02
Fstl3	Follistatin-like 3	0.6487	2.3288E-02
Tst	Thiosulfate sulfurtransferase, mitochondrial	0.6262	4.6175E-02
1700052K11Rik	RIKEN cDNA 1700052 K11 gene	0.6244	2.8782E-02
6030443J06Rik	RIKEN cDNA 6030443 J06 gene	0.6068	4.4283E-02
Npnt	Nephronectin	0.6065	1.4660E-02
Tbc1d7	TBC1 domain family, member 7	0.5949	4.5364E-02
15 downregulated genes			
Noct	Nocturnin	− 2.1807	4.9148E-03
Arrdc2	Arrestin domain containing 2	− 1.2999	3.1015E-03
Piga	Phosphatidylinositol glycan anchor biosynthesis, class A	− 1.0936	4.6036E-02
Ppara	Peroxisome proliferator activated receptor alpha	− 0.9904	3.0533E-02
Dusp7	Dual specificity phosphatase 7	− 0.8921	4.6373E-03
Ip6k2	Inositol hexaphosphate kinase 2	− 0.8573	3.8128E-02
Bcl2l1	BCL2-like 1	− 0.7134	2.2437E-02
Fam126b	Family with sequence similarity 126, member B	− 0.7033	4.0931E-02
Cdc37l1	Cell division cycle 37-like 1	− 0.6873	4.5256E-02
Auts2	Autism susceptibility candidate 2	− 0.6657	2.6343E-02
Nfkbia	Nuclear factor of kappa light polypeptide gene enhancer in B cells inhibitor, alpha	− 0.6497	4.7352E-02
1700016C15Rik	RIKEN cDNA 1700016C15 gene	− 0.6494	2.7854E-02
Klf13	Kruppel-like factor 13	− 0.6364	1.0617E-02
Ccng2	Cyclin G2	− 0.6010	4.8169E-02
Itch	Itchy, E3 ubiquitin protein ligase	− 0.5971	2.3641E-02

Fold change is displayed as log^2^ value

**Fig. 6:**
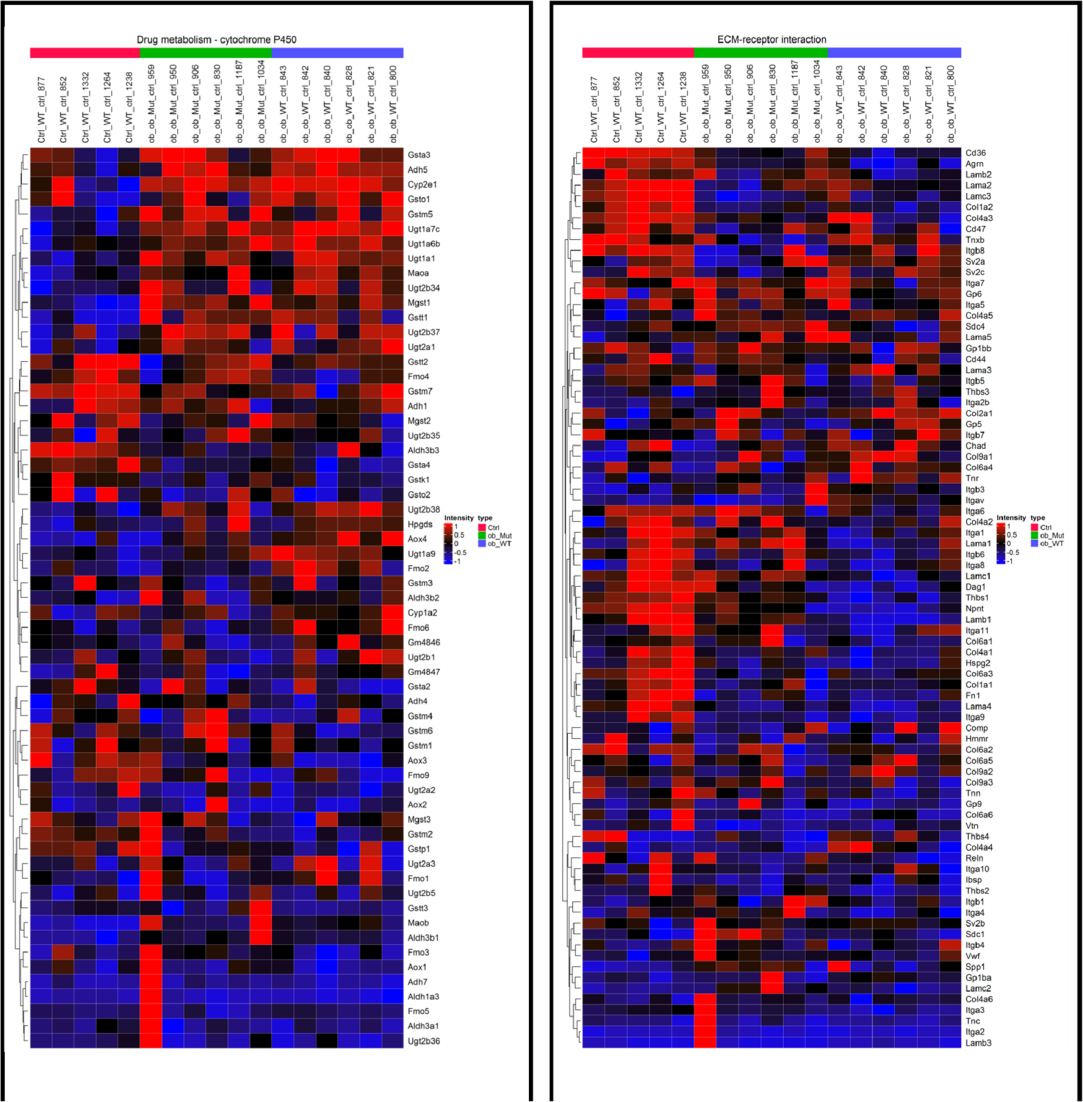
Gene set enrichment analysis was performed on the dataset. From the significantly enriched pathways, the most upregulated and downregulated according to normalized enrichment score were selected to be shown on a heat map

Gene set enrichment analysis (GSEA) revealed 15 pathways to be significantly enriched in ob/ob mice as compared to WT (6 upregulated, 9 downregulated; normalized enrichment scores (NES) range: − 2.46 to 1.79; *P*_adj_ < 0.05) (Supplementary table 2). The most upregulated (drug metabolism—cytochrome P450) and the most downregulated pathways (ECM-receptor interaction) are depicted in Fig. 6 as heat maps. Of the 15 enriched pathways found in the comparison ob/ob vs. WT mice, 4 pathways were also significantly upregulated in hCN1 TG ob/ob mice, i.e., cell adhesion molecules (CAMs) (*P*_adj_ = 0.01, NES = 1.8), ECM-receptor interaction (*P*_adj_ = 0.03, NES = 1.7), focal adhesion (*P*_adj_ = 0.02, NES = 1.54), and Rap1 signaling (*P*_adj_ = 0.03, NES = 1.5) (Supplementary table 3).

## Discussion

In the present study, we, for the first time, provide evidence that serum CN1 expression aggravates DKD, reflected by an increased ACR and more severe renal histology.

We and others have previously reported that the CNDP1 (CTG)_n_ polymorphism is associated with susceptibility to develop DKD in patients with type 2 diabetes. The shortest allelic form, associated with low CN1 enzymatic activities and low serum CN1 concentrations, is more common in patients without nephropathy. Yet, there is also a fair amount of controversy on the role of CN1 for developing DKD as other studies failed to replicate these findings in cohorts of different ethnicities or in patients with type 1 diabetes [21]. Although the study of Sauerhöfer et al. [12] in human CN1 overexpressing db/db mice revealed aggravated diabetes, it failed to show an effect on DKD. This might be explained by the fact that db/db mice only develop mild renal damage, which could mask a possible influence of a disease modifier such as CN1. In contrast to db/db mice, novel models such as the BTBR^Ob/Ob^ mice develop a more severe DKD phenotype with profound albuminuria, which, compared with nondiabetic WT mice, is equivalent to a 10- to 20-fold increase in ACR [22]. Clearly, this makes the model more robust in terms of renal endpoints and hence more suitable to study the relative influence of CN1 on DKD.

Plasma and renal carnosine concentrations were strongly diminished in hCN1 TG mice. Since carnosine has been demonstrated to have several beneficial effects on DKD such as ROS-/RCS-scavenging and antioxidative properties, depletion of carnosine may lead to an increased formation of AGE products and therefore might be accountable for the disease aggravating effect in hCN1 TG mice [13, 15–17, 23, 24]. Although we could not show an increase of glyoxal or methylglyoxal in our model, we detected a significantly higher concentration of 3-deoxyglucosone in the plasma of transgenic diabetic mice. An increased level of 3-deoxyglucosone has also been detected in patients with diabetic kidney disease compared with patients with diabetes alone [25, 26]. Yet, protein carbonylation in renal tissue did not differ between diabetic and nondiabetic mice. Based on previous studies, in which we showed a role for carnosine in the clearance of acrolein [16] and studies performed by others using carnosine or carnosine analog to prevent the formation of 4-hydroxynonenal [27], it was expected that depletion of carnosine in hCN1 TG ob/ob mice would have a more substantial impact on aldehyde stress [10, 11, 27]. Since carnosine seems to increase insulin sensitivity [28], this better explains the aggravating effect of serum carnosinase 1 on DKD. The assumption that carnosine influences insulin sensitivity is further corroborated by our findings that fasting plasma glucose is increased in hCN1 TG ob/ob mice despite comparable plasma levels of insulin. Our data also suggest that carnosine is not essential for insulin secretion, albeit that carnosine supplementation is able to support insulin secretion of pancreatic islets in this model [16].

Although we assessed carnosine and anserine concentration only in nondiabetic hCN1 TG WT and not in diabetic hCN1 TG ob/ob mice, findings of Peters et al. [29] and Riedl et al. [30] that CN1 activity is upregulated by reactive carbonyl- and oxygen species (RCS and ROS) through posttranslational modifications suggest that renal carnosine concentrations are even lower in diabetic hCN1 TG ob/ob compared with WT diabetic ob/ob mice.

hCN1 TG ob/ob mice showed a decreased body weight compared with their nonTG ob/ob littermates. In the work of Sauerhöfer et al. [12], similar findings were made for hCN1 TG db/db mice. This may be caused by increased glucosuria in hCN1 TG mice.

Recently Chittka et al. [31] have reported on differences in glomerular gene expression profiles between diabetic and nondiabetic BTBR mice in a time-resolved manner. Since we used the renal cortex instead of morphologically dissected glomeruli, this makes direct comparisons difficult. Nonetheless, a number of the reported differentially expressed genes were also found in our data set, e.g., Hdc, Hyal, Hmgcs2, C3, albeit that the total number of DEGs we found was significantly lower than in the study of Chittka et al. [31]. Although this is partly due to the use of different arrays (Mouse Whole Transcriptome 1.0 ST array vs. Mouse Gene 2.0 ST array), there remains a large difference in the number of DEGs between both studies even when considering only coding DEG (1044 vs. 297). Even though renal and serum parameters, as well as some of the histological features in renal tissue, were worse in hCN1 TG mice, the number of DEGs was limited to a subset of 26 genes with a FC-threshold of > 1.5. We cannot, however, exclude that the differences in gene expression profile between diabetic TG and nonTG mice would have been larger if glomeruli were analyzed selectively as previously reported by Chittka et al. [31].

Amongst the 26 DEG in diabetic hCN1 TG mice, many have been reported to be pivotal in the pathogenesis of DKD. In an independent experiment, however, significance between hCN1 TG ob/ob and ob/ob could not be confirmed by qPCR, albeit that for most of these genes, the direction of change was similar to the Affymetrix data set. The considerable variation in gene expression, the relative low fold-change, and the relatively low number of animals (*n* = 6 per group) in the independent confirmatory experiment may explain why significance was not reached. The two genes that showed the largest change in hCN1 TG ob/ob compared with nonTG ob/ob mice in the Affymetrix data set, i.e., nocturnin (Noct) and arrestin domain-containing protein 2 (Arrdc2), were also significantly changed in the independent confirmatory experiment. Noct is a circadian protein that regulates the cellular transcriptome via control of poly(A) tail length of RNA transcripts [32]. Why renal Noct is strongly downregulated in hCN1 TG ob/ob mice, is currently not known. Our observation that carnosine levels in cerebral tissue were significantly reduced in hCN1 TG poses the question as to whether this is also true for homocarnosine, another substrate of CN1. Because homocarnosine is considered as a reservoir for GABA, increased CN1 activity may affect GABA homeostasis. This, in turn, may affect the central circadian pacemaker of the suprachiasmatic nuclei (SCN) in the brain, which uses GABA as a principal neurotransmitter [33]. Although further experiments are warranted to substantiate this assumption, it is worth to mention that Noct regulates metabolic adaptation in brown adipose tissue [34] and that it may maintain a proper metabolic balance in the face of metabolic challenges [35]. Hence, the downregulation of Noct in obese diabetic may further dysregulate metabolism in BTBR^ob/ob^ mice.

In conclusion, this study demonstrates that the hCN1 TG BTBR^ob/ob^ (ob/ob) mice develop more severe DKD. Despite this, the influence of the transgene on the renal transcriptome is limited. It needs to be assessed if the downregulation of Noct in diabetic animals contributes to this phenotype. Likewise, the effect of serum CN-1 expression on homocarnosine concentrations in cerebral tissue and its relation to Noct expression should be addressed to better understand if and how CN1 affects metabolic processes in these mice.

## Electronic supplementary material

ESM 1 (DOCX 2119 kb).

ESM 2 (XLSX 525 kb).

ESM 3 (XLSX 57 kb).

## Data Availability

The microarray data can be found in the supplementary data.

## Abbreviations

CN1: Carnosinase 1
CN2: Carnosinase 2
CKD: Chronic kidney disease
DKD: Diabetic kidney disease
TG: Transgenic
SD: Standard deviation
