# Supplementary material for: Human carnosinase 1 overexpression aggravates diabetes and renal impairment in BTBROb/Ob mice
Source: J Mol Med (Berl). 2020 Aug 15;98(9):1333–46. doi: 10.1007/s00109-020-01957-0 (PMC7447680; doi:10.1007/s00109-020-01957-0)
Supplement: Supplementary file 1 — (DOCX 2119 kb). [file 109_2020_1957_MOESM1_ESM.docx]

**Supplementary data for**

**Human carnosinase 1 overexpression aggravates diabetes and renal impairment in BTBR^Ob/Ob^ mice**

Jiedong Qiu^1+ #^, Thomas Albrecht ^1+^, Shiqi Zhang^1, 6+^, Sibylle J. Hauske^1^, Angelica Rodriguez-Niño^1^, Xinmiao Zhang^1^, Darya Nosan^1^, Diego O. Pastene^1^, Carsten Sticht^2^, Carolina Delatorre^2^, Harry van Goor^3^, Stefan Porubsky^4^, Bernhard K. Krämer^1, 5^, Benito A. Yard^1, 5^

^1^ 5^th^ Medical Department, University Hospital Mannheim, Heidelberg University, Mannheim, Germany

^2^ Central Medical Research Facility ZMF, University Hospital Mannheim, Heidelberg University, Mannheim, Germany

^3^ Department of Pathology and Medical Biology, University Medical Centre Groningen and University of Groningen, Groningen, the Netherlands

^4^ Institute of Pathology, University Hospital Mannheim, Heidelberg University, Mannheim, Germany

^5^ European Center for Angioscience, Mannheim, Germany

^6^ Department of Endocrinology, The first affiliated hospital of Anhui Medical University

^#^jiedong.qiu@medma.uni-heidelberg.de, Phone: +49 621 383-2937

^+^these authors contributed equally to this work

Contents

[Supplementary Methods 3](#_Toc42692899)

[CN1 concentration assay 3](#_Toc42692900)

[CN1 activity assay 4](#_Toc42692901)

[Supplementary Figures 5](#_Toc42692902)

[Supplementary Tables 7](#_Toc42692903)

[Supplementary Table 1: Expression influenced by both diabetes and the TG 7](#_Toc42692904)

[Supplementary Table 2: GSEA 8](#_Toc42692905)

[Supplementary Table 3: GSEA common hits of both comparisons 9](#_Toc42692906)

# Supplementary Methods

## CN1 concentration assay

CN1 concentrations in serum and urine were measured by an in-house-made ELISA as described previously ^20,50^. In short, high-absorbent microtiter plates (Greiner Frickenhausen, Germany) were coated overnight with 100 μl 10 μg/ml goat polyclonal anti-human CN1 (R&D Wiesbaden, Germany). Hereafter, the plates were washed and incubated with 0.05% of milk powder to block unspecific binding to the plate. Serum samples were tested in appropriate dilutions from 1:50-1:200. All samples were tested in duplicates. The plates were placed on a shaker for 1 h with 300rpm and subsequently washed with PBS containing 0,1% Tween. Rabbit polyclonal anti-CNDP1 antibody (ATLAS/Abcam Cambridge, United Kingdom) was added for 1h followed by extensive washing. Goat anti-rabbit HRP-conjugated IgG antibody (Santa Cruz, USA) was added for 30 min. Deep-blue peroxidase (Roche Diagnostics Mannheim, Germany) was used for colour development and the reaction was stopped after 10 min by the addition of 50μl of 1M H_2_SO_4_. Optical density at 450nm (OD450) was measured on a Tecan infinite 2000 ELISA reader. A serial dilution of pooled serum with known carnosinase concentration was used as the standard. CN1 concentrations were assessed in the linear part of the dilution curve with a lower sensitivity of 31 ng/ml. Readings below the threshold were retested in a lower concentration and then if still under the detection limit considered as undetectable. On each ELISA and for each test, PBS was included as a blank value.

## CN1 activity assay

CN1 activity was measured using a modified method described previously by Teufel et al. ^18^. Briefly, carnosine dissolved in 50mM pH=7,5 Tris buffer (both from Sigma-Aldrich Germany) was added to serum to an end concentration of 0,2 mg/ml (880µM) on ice. The final solution was divided into three aliquots. Enzym activity in the first tube was immedieatly blocked by addition of 10% sulfosalicylic acid (Sigma-Aldrich Germany) in a dilution of 1:5. The remaining samples were incubated for 5min respectively 15min at 30° C before the addition of sulfosalicylic acid. After addition of sulfosalicylic all samples were immediately incubated for 30min on ice followed by centrifugation at 12000g for 5min. The supernatants were mixed with an equal volume of a solution containing 50mM of Tris buffer (pH=7,5), 1% trichloroacetic acid in distilled water and 5mg/ml o-phtaldialdehyd in 2N NaOH (Sigma-Aldrich Germany) for derivatization of the released histidine and incubated for 30min at 30° C. A serial dilution with known histidine concentrations were treated similar and used as standard curve. Hereafter, fluorescence was measured at excitation - emission wavelengths of 360nm and 465nm respectively. Enzyme activity was calculated using a linear regression curve based on the three time points (0min, 5min and 15min).

# Supplementary Figures

**Supplementary Figure 1**: **a)** Dicarbonyls from BTBR^Ob/Wt^ (WT), non-transgenic BTBR^Ob/Ob^ (ob/ob) and transgenic BTBR^Ob/Ob^ (TG ob/ob) were assessed (n = 4 per group) in serum (upper panels) and renal tissue (lower panels). The data are depicted as mean ± SD. One-way ANOVA followed by Tukey post-hoc test was used to compare the groups. * for p-values < 0.05, *** for p-values < 0.001 and n.s. for p-values >0.05. **b)** Oxyblot analysis was conducted to analyze the renal protein carbonylation.


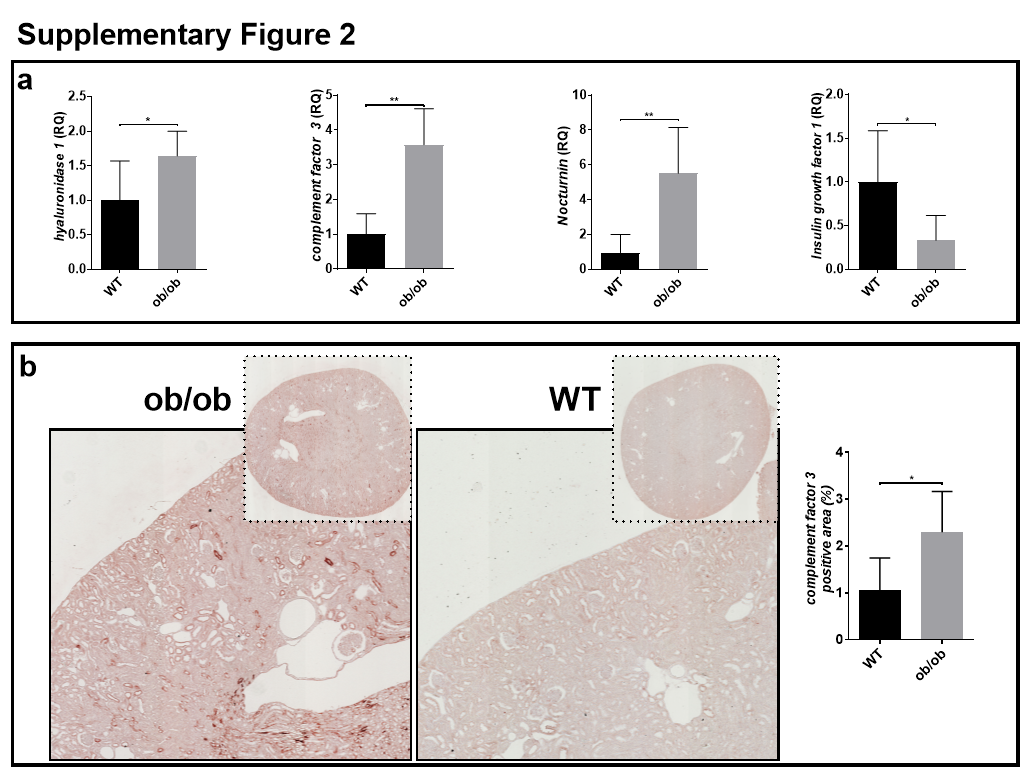


**Supplementary Figure 2**: **a)** For confirmation of the Affymetrix data set, RNA was isolated from kidneys of an independent group of non-diabetic BTBR^Ob/Wt^ (WT) and diabetic BTBR^Ob/Ob^ (ob/ob) mice. qPCR analysis was performed on 4 randomly chosen genes (Hyal1, C3, Noc and Igf1) belonging to the top 15 of changed expression levels. * for p-values < 0.05, ** for p-values < 0.01 and n.s. for p-values >0.05. **b)** C3 protein was assessed by immunohistochemistry (panels to the left and in the middle) and quantified by morphometric analysis (panel to the right).

# Supplementary Tables

| Supplementary Table 1: Expression influenced by both diabetes and the TG **5 up-regulated genes** | | | |  |  |
| --- | --- | --- | --- | --- | --- |
| **Gene symbol** | **Gene name** | **Fold-change (OB vs WT)** | ***p-Value*** | **Fold-change (OB TG vs OB)** | ***p-Value*** |
| Egfl6 | EGF-like-domain, multiple 6 | -1.7557 | 1.19E-03 | 0.9664 | 3.60E-02 |
| Thbs1 | thrombospondin 1 | -1.0510 | 4.27E-03 | 0.6836 | 4.38E-02 |
| Fstl3 | follistatin-like 3 | -0.6451 | 2.37E-02 | 0.6487 | 2.33E-02 |
| Tst | thiosulfate sulfurtransferase, mitochondrial | -0.7436 | 2.19E-02 | 0.6262 | 4.62E-02 |
| Npnt | nephronectin | -1.0892 | 3.68E-04 | 0.6065 | 1.47E-02 |
|  |  |  |  |  |  |
| **12 down-regulated genes** | | | |  |  |
| **Gene symbol** | **Gene name** | **Fold-change (OB vs WT)** | ***p-Value*** | **Fold-change (OB TG vs OB)** | ***p-Value*** |
| Noct | nocturnin | 2.444795 | 2.24E-03 | -2.18066 | 4.91E-03 |
| Arrdc2 | arrestin domain containing 2 | 1.309515 | 2.94E-03 | -1.29994 | 3.10E-03 |
| Piga | phosphatidylinositol glycan anchor biosynthesis, class A | 1.106852 | 4.44E-02 | -1.09358 | 4.60E-02 |
| Dusp7 | dual specificity phosphatase 7 | 0.931644 | 3.48E-03 | -0.89208 | 4.64E-03 |
| Ip6k2 | inositol hexaphosphate kinase 2 | 0.951786 | 2.38E-02 | -0.85725 | 3.81E-02 |
| Bcl2l1 | BCL2-like 1 | 0.90422 | 5.65E-03 | -0.71336 | 2.24E-02 |
| Fam126b | family with sequence similarity 126, member B | 0.804612 | 2.19E-02 | -0.70335 | 4.09E-02 |
| Cdc37l1 | cell division cycle 37-like 1 | 0.814942 | 2.12E-02 | -0.68728 | 4.53E-02 |
| Auts2 | autism susceptibility candidate 2 | 0.834902 | 7.33E-03 | -0.66568 | 2.63E-02 |
| Nfkbia | nuclear factor of kappa light polypeptide gene enhancer in B cells inhibitor, alpha | 0.838544 | 1.45E-02 | -0.64967 | 4.74E-02 |
| 1700016C15Rik | RIKEN cDNA 1700016C15 gene | 0.952867 | 2.86E-03 | -0.64939 | 2.79E-02 |
| Itch | itchy, E3 ubiquitin protein ligase | 0.713436 | 8.75E-03 | -0.5971 | 2.36E-02 |
|  | ***Fold change*** *is displayed as Log2 value* | | | | |

| Supplementary Table 2: GSEA **OB vs WT** | | |
| --- | --- | --- |
| **KEGG pathway code - name** | **NES OB vs WT** | ***p*-Value** |
| [04512 - ECM-receptor interaction](http://www.genome.jp/kegg-bin/show_pathway?map=mmu04512&show_description=show) | -2.46 | 0.02 |
| 01040 - Biosynthesis of unsaturated fatty acids | -2.08 | 0.04 |
| 00100 - Steroid biosynthesis | -1.91 | 0.05 |
| 05218 - Melanoma | -1.75 | 0.04 |
| 04640 - Hematopoietic cell lineage | -1.73 | 0.04 |
| 04510 - Focal adhesion | -1.62 | 0.02 |
| 04015 - Rap1 signaling pathway | -1.59 | 0.02 |
| 04514 - Cell adhesion molecules (CAMs) | -1.57 | 0.04 |
| 04151 - PI3K-Akt signaling pathway | -1.4 | 0.05 |
| 04740 - Olfactory transduction | 1.34 | 0.02 |
| 04010 - MAPK signaling pathway | 1.52 | 0.04 |
| 03013 - RNA transport | 1.57 | 0.04 |
| 04141 - Protein processing in endoplasmic reticulum | 1.73 | 0.02 |
| 00983 - Drug metabolism - other enzymes | 1.75 | 0.04 |
| 00982 - Drug metabolism - cytochrome P450 | 1.79 | 0.04 |
|  |  |  |
| **OB CN+ vs OB CN-** |  |  |
| **KEGG pathway code - name** | **NES OB CN- vs OB CN+** | ***p*-Value** |
| 05206 - MicroRNAs in cancer | 1.5 | 0.01 |
| 04015 - Rap1 signaling pathway | 1.5 | 0.03 |
| 04510 - Focal adhesion | 1.54 | 0.02 |
| 05205 - Proteoglycans in cancer | 1.63 | 0.01 |
| 04611 - Platelet activation | 1.63 | 0.02 |
| 04810 - Regulation of actin cytoskeleton | 1.67 | 0.01 |
| 04512 - ECM-receptor interaction | 1.7 | 0.03 |
| 03008 - Ribosome biogenesis in eukaryotes | 1.72 | 0.03 |
| 05210 - Colorectal cancer | 1.74 | 0.02 |
| 04670 - Leukocyte transendothelial migration | 1.77 | 0.01 |
| 03040 - Spliceosome | 1.81 | 0.01 |
| 05212 - Pancreatic cancer | 1.83 | 0.01 |
| 04514 - Cell adhesion molecules (CAMs) | 1.83 | 0.01 |
| 05144 - Malaria | 1.89 | 0.02 |
| 05219 - Bladder cancer | 1.9 | 0.02 |

| Supplementary Table 3: GSEA common hits of both comparisons | | | | | |
| --- | --- | --- | --- | --- | --- |
| **KEGG pathway code - name** | **NES OB vs WT** | | ***p*-Value** | **NES OB CN- vs OB CN+** | ***p*-Value** |
| [04514 - Cell adhesion molecules (CAMs)](http://www.genome.jp/kegg-bin/show_pathway?map=mmu04514&show_description=show) | | -1.57 | 0.04 | 1.83 | 0.01 |
| [04512 - ECM-receptor interaction](http://www.genome.jp/kegg-bin/show_pathway?map=mmu04512&show_description=show) | -2.46 | | 0.02 | 1.7 | 0.03 |
| [04510 - Focal adhesion](http://www.genome.jp/kegg-bin/show_pathway?map=mmu04510&show_description=show) | -1.62 | | 0.02 | 1.54 | 0.02 |
| [04015 - Rap1 signaling pathway](http://www.genome.jp/kegg-bin/show_pathway?map=mmu04015&show_description=show) | -1.59 | | 0.02 | 1.5 | 0.03 |
